# Challenges Conveying Clinical Equipoise and Exploring Patient Treatment Preferences in an Oncology Trial Comparing Active Monitoring with Radiotherapy (ROAM/EORTC 1308)

**DOI:** 10.1634/theoncologist.2019-0571

**Published:** 2020-02-11

**Authors:** Frances C. Sherratt, Stephen L. Brown, Brian J. Haylock, Priya Francis, Helen Hickey, Carrol Gamble, Michael D. Jenkinson, Bridget Young

**Affiliations:** ^1^ Institute of Population Health Sciences, University of Liverpool United Kingdom; ^2^ Liverpool Clinical Trials Centre, University of Liverpool United Kingdom; ^3^ Institute of Translational Medicine, University of Liverpool United Kingdom; ^4^ Clatterbridge Cancer Centre Wirral United Kingdom; ^5^ The Walton Centre NHS Foundation Trust Liverpool United Kingdom

**Keywords:** Clinical trial, Communication, Clinical equipoise, Qualitative, Neuro‐oncology, Radiotherapy

## Abstract

**Introduction:**

Providing balanced information that emphasizes clinical equipoise (i.e., uncertainty regarding the relative merits of trial interventions) and exploring patient treatment preferences can improve informed consent and trial recruitment. Within a trial comparing adjuvant radiotherapy versus active monitoring following surgical resection for an atypical meningioma (ROAM/EORTC‐1308), we explored patterns in communication and reasons why health practitioners may find it challenging to convey equipoise and explore treatment preferences.

**Materials and Methods:**

Qualitative study embedded within ROAM/EORTC‐1308. Data were collected on 40 patients and 18 practitioners from 13 U.K. sites, including audio recordings of 39 patients’ trial consultations, 23 patient interviews, and 18 practitioner interviews. Qualitative analysis drew on argumentation theory.

**Results:**

Practitioners acknowledged the importance of the research question that the trial aimed to answer. However, they often demonstrated a lack of equipoise in consultations, particularly with eligible patients who practitioners believed to be susceptible to side effects (e.g., cognitive impairment) or inconvenienced by radiotherapy. Practitioners elicited but rarely explored patient treatment preferences, especially if a patient expressed an initial preference for active monitoring. Concerns about coercing patients, loss of practitioner agency, and time constraints influenced communication in ways that were loaded against trial participation.

**Conclusions:**

We identified several challenges that practitioners face in conveying equipoise and exploring patient treatment preferences in oncology, and particularly neuro‐oncology, trials with distinct management pathways. The findings informed communication about ROAM/EORTC‐1308 and will be relevant to enhancing trial communication in future oncology trials. Qualitative studies embedded within trials can address difficulties with communication, thus improving informed consent and recruitment. ROAM/EORTC‐1308 RCT: ISRCTN71502099.

**Implications for Practice:**

Oncology trials can be challenging to recruit to, especially those that compare treatment versus monitoring. Conveying clinical equipoise and exploring patient treatment preferences can enhance recruitment and patient understanding. This study focused on the challenges that practitioners encounter in trying to use such communication strategies and how practitioners may inadvertently impede patient recruitment and informed decision making. This article provides recommendations to support practitioners in balancing the content and presentation of trial management pathways. The results can inform training to optimize communication, especially for neuro‐oncology trials and trials comparing markedly different management pathways.

## Abstract 2

Recruitment of enough eligible patients to randomized controlled clinical trials can be challenging. Within the context of an active clinical trial, this study explored communication strategies, especially how practitioners conveyed equipoise, explored and responded to patient treatment preferences, and the challenges encountered.

## Introduction

Randomized controlled trials frequently struggle to recruit adequate numbers of eligible patients 1, 2. Poor recruitment can compromise a trial's statistical power and generalizability 3, deny or delay the benefits of health research for future patients 4, and increase financial costs 1, 2. Patient understanding and acceptance of trial components and principles that directly affect their care is also fundamental to recruitment and informed consent 5.

Recruitment into trials that include markedly different management pathways can be particularly challenging because of patient and practitioner treatment preferences 6. A common preventable reason for poor trial recruitment is the absence of “clinical equipoise” among health practitioners 7, where clinical equipoise is defined as uncertainty about the relative clinical merits of the intervention arms in a trial 8. Practitioners may knowingly or unknowingly communicate positive or negative beliefs about the effectiveness or safety of either treatments being investigated, which can undermine patient willingness to be randomized 9.

Patients may also have unfounded beliefs about trial treatments that can deter them from participating or lead to suboptimally informed decision making. Exploring patient treatment preferences during trial consultations is advocated as a way to improve patient decision making and avoid unfounded beliefs about treatments deterring them from being randomized 10, 11. It entails eliciting and acknowledging the reasons that underlie treatment preferences, providing information to balance preferences and address any misapprehensions, and emphasizing the importance of keeping an open mind about trial treatments 11.

Qualitative studies have been embedded in trials to identify and address recruitment challenges 12, 13, 14, 15, 16, 17. These studies examine how practitioners communicate about trials, before providing tailored feedback to support them in optimizing informed consent discussions and recruitment 18. Previous qualitative studies have identified communication issues in trials and supported practitioners to balance the content and presentation of information on trial treatments, to emphasize clinical equipoise, and optimize informed consent and recruitment 19. Such studies advocate that trial practitioners should present equipoise convincingly 19 and explore treatment preferences with patients 10, 11.

The Radiation versus Observation for Atypical Meningioma (ROAM/EORTC‐1308) trial compares adjuvant radiotherapy with active monitoring following complete surgical resection of an atypical meningioma 20. As the trial compares two markedly different management pathways—active treatment (radiotherapy) to nonactive treatment (active monitoring)—we anticipated that recruitment would be challenging. It is currently unclear whether practitioners recruiting to oncology trials, such as ROAM/EORTC‐1308, are adopting trial communication strategies advocated in the literature and what the challenges are in doing so. This qualitative study embedded in ROAM/EORTC‐1308 examined how practitioners conveyed equipoise, how they explored and responded to patient treatment preferences, and the challenges they encountered. As part of the study we provided practitioners with feedback as the trial was ongoing in order to enhance their communication and thereby optimize informed consent and recruitment. The analyses we report here describe practitioners’ communication about the trial, focusing on their efforts to convey equipoise and to manage patients’ treatment preferences.

## Materials and Methods

### Overview

**Figure 1:**
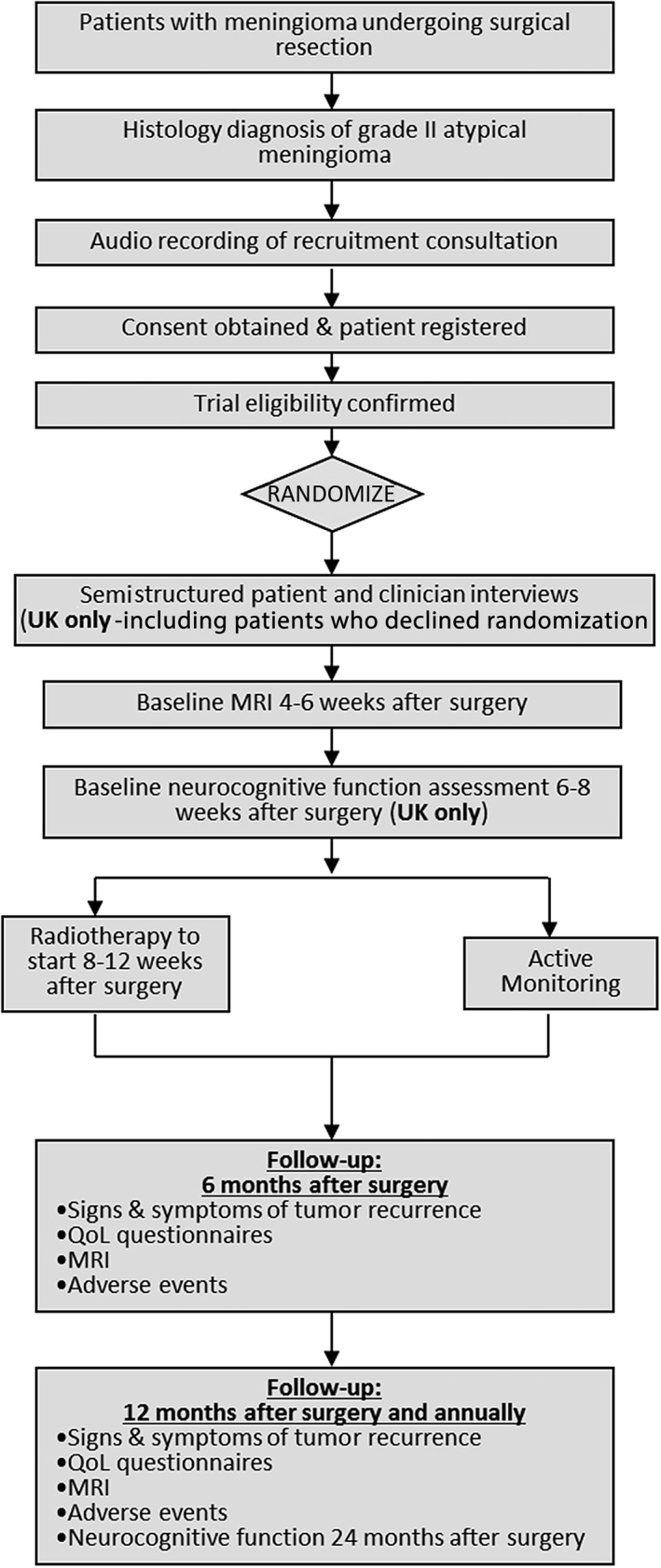
Summary of the design of ROAM/EORTC‐1308 and the embedded qualitative study.Abbreviations: MRI, magnetic resonance imaging; QoL, quality of life.

The design of ROAM/EORTC‐1308 and the embedded qualitative study is summarized in Figure 1. Briefly, it is an ongoing multicenter, international trial, recruiting patients who have undergone gross total resection of intracranial atypical meningioma and randomizing them to either active monitoring with magnetic resonance imaging (MRI) or 6 weeks of adjuvant radiotherapy (60 Gy in 30 fractions). The trial hypothesis is that adjuvant radiotherapy reduces the risk of meningioma recurrence compared with active monitoring. The primary outcome is time to MRI evidence of tumor recurrence (i.e., progression‐free survival), and secondary outcomes include toxicity of radiotherapy, quality of life, neurocognitive function, time to second line treatment, time to death, and incremental cost per quality‐adjusted life year gained 20.

We used established qualitative methodology 18, 21 to analyze trial consultation recordings and semistructured interviews with patients and practitioners. Additionally, we used findings from the ongoing analysis of these data to inform feedback to practitioners (delivered via written summaries tailored to individual practitioners, two U.K.‐based and one European workshop, one U.K. and Europe webinar, and a “hints and tips” feedback sheet distributed to all trial sites) to help them enhance their communication, although we did not formally evaluate the impact of this feedback. A U.K. National Research Ethics Service Committee (15/NE/0013) approved ROAM/EORTC‐1308 and the embedded qualitative study.

### Setting and Participants

ROAM/EORTC‐1308 opened in April 2016. Recruitment is ongoing in 49 sites across the U.K., Europe, and Australia. The qualitative study was conducted in 13 of 20 U.K. sites and ran from April 2016 to November 2018. Practitioners (neurosurgeons, radiation oncologists, and research nurses) invited eligible patients to participate in ROAM/EORTC‐1308 typically 2–5 weeks after surgery. Patients eligible for ROAM/EORTC‐1308 were also eligible for the qualitative study; they could participate in the trial, the qualitative study, both, or neither. Patients and practitioners who participated in the qualitative study agreed for their trial consultation to be recorded or completed an interview, or both. Our purposive sampling strategy aimed for diversity in patient age, gender, socio‐economic status, hospital site, and practitioner role and to include patients who declined ROAM/EORTC‐1308 and those who consented.

### Procedure

#### *Consultation Recordings*

Patients attended clinic consultations at which practitioners typically explained the meningioma pathology results before discussing ROAM/EORTC‐1308. Practitioners requested the permission of patients to audio record the trial consultation and, if the patient agreed, obtained written consent at the end of the consultation. Some patients who expressed an interest in ROAM/EORTC‐1308 were invited to attend a second consultation, which was also audio recorded where possible. Research nurses forwarded copies of the qualitative study consent form to the study researcher (F.C.S.) and uploaded consultation recordings via a secure electronic facility.

#### *Patient and Health Professional Interviews*

F.C.S. (a qualitative researcher with a background in health research) conducted and audio recorded semistructured interviews with patients and practitioners, either face‐to‐face or by telephone, after first obtaining their consent. Interviews were topic guided (supplemental online Appendix A) but adapted over the course of the analysis to explore relevant issues. Table 1 summarizes the topics explored in the patient and practitioner interviews. Where an audio‐recorded trial consultation was available, F.C.S. reviewed this before the interview to tailor questions to participants and explore pertinent aspects of communication.

**Table 1:**
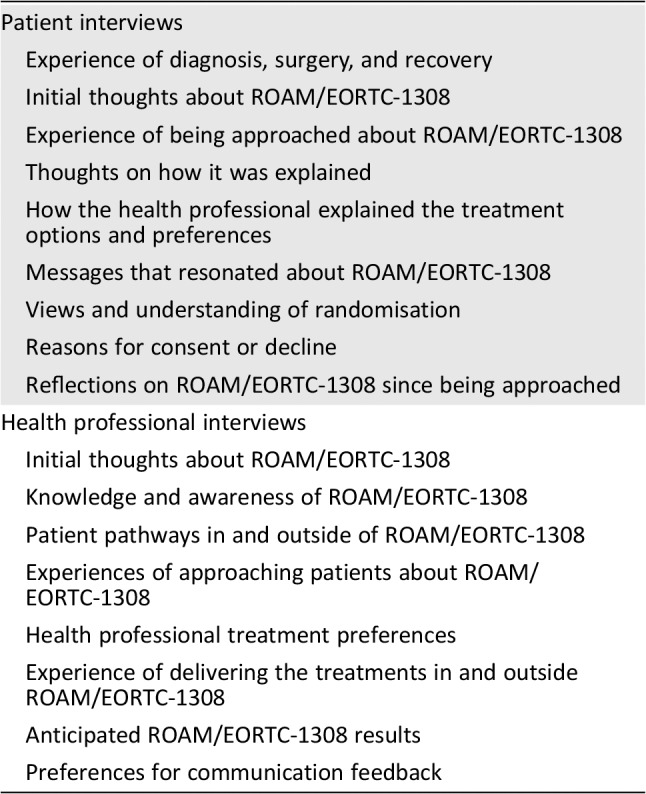
Overview of the topics explored in the patient and health professional interviews

Patient interviews
Experience of diagnosis, surgery, and recovery
Initial thoughts about ROAM/EORTC‐1308
Experience of being approached about ROAM/EORTC‐1308
Thoughts on how it was explained
How the health professional explained the treatment options and preferences
Messages that resonated about ROAM/EORTC‐1308
Views and understanding of randomisation
Reasons for consent or decline
Reflections on ROAM/EORTC‐1308 since being approached
Health professional interviews
Initial thoughts about ROAM/EORTC‐1308
Knowledge and awareness of ROAM/EORTC‐1308
Patient pathways in and outside of ROAM/EORTC‐1308
Experiences of approaching patients about ROAM/EORTC‐1308
Health professional treatment preferences
Experience of delivering the treatments in and outside ROAM/EORTC‐1308
Anticipated ROAM/EORTC‐1308 results
Preferences for communication feedback

### Qualitative Analysis

All consultation recordings and interviews were transcribed and anonymized. Data collection continued until data saturation was reached, the point at which new themes ceased being identified 22. Data analysis was generally interpretive and pluralistic, drawing on thematic analysis 23 and the framework approach 24. Analysis of consultations was informed by argumentation theory 25, whereby we examined the content of arguments made by practitioners in informing patients and the arguments made by patients in justifying their preferences. This allowed us to identify patterns in communication relevant to clinical equipoise and treatment preferences (see example analysis excerpt from one consultation in Table 2). Although this initially involved F.C.S. focusing on segments of consultation text and comparing these within and between transcripts, we also examined the data more holistically to make sure our interpretations of the segments fitted with the consultation as a whole. F.C.S. also listened to audio recordings of consultations to take account of subtleties such as the tone and pace of speech.

**Table 2:**
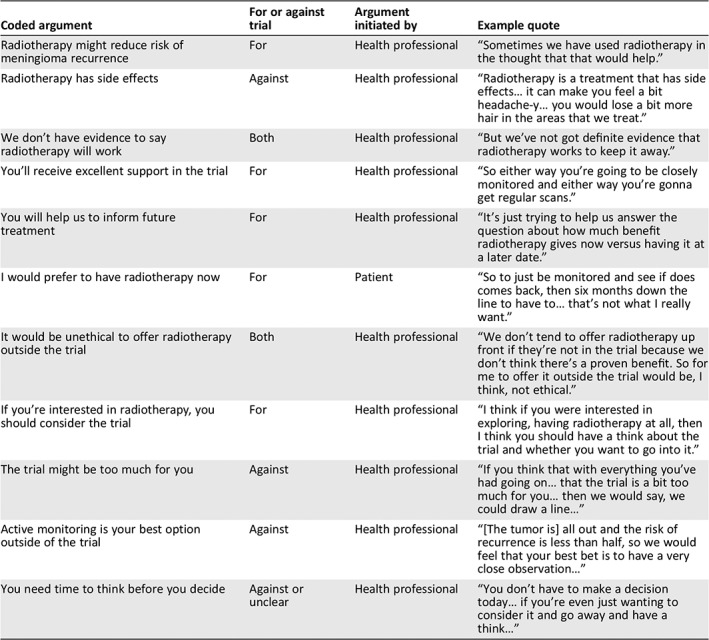
Excerpt of analysis from one patient's ROAM/EORTC‐1308 consultation, applying argumentation theory

Coded argument	For or against trial	Argument initiated by	Example quote
Radiotherapy might reduce risk of meningioma recurrence	For	Health professional	“Sometimes we have used radiotherapy in the thought that that would help.”
Radiotherapy has side effects	Against	Health professional	“Radiotherapy is a treatment that has side effects… it can make you feel a bit headache‐y… you would lose a bit more hair in the areas that we treat.”
We don't have evidence to say radiotherapy will work	Both	Health professional	“But we've not got definite evidence that radiotherapy works to keep it away.”
You'll receive excellent support in the trial	For	Health professional	“So either way you're going to be closely monitored and either way you're gonna get regular scans.”
You will help us to inform future treatment	For	Health professional	“It's just trying to help us answer the question about how much benefit radiotherapy gives now versus having it at a later date.”
I would prefer to have radiotherapy now	For	Patient	“So to just be monitored and see if does comes back, then six months down the line to have to… that's not what I really want.”
It would be unethical to offer radiotherapy outside the trial	Both	Health professional	“We don't tend to offer radiotherapy up front if they're not in the trial because we don't think there's a proven benefit. So for me to offer it outside the trial would be, I think, not ethical.”
If you're interested in radiotherapy, you should consider the trial	For	Health professional	“I think if you were interested in exploring, having radiotherapy at all, then I think you should have a think about the trial and whether you want to go into it.”
The trial might be too much for you	Against	Health professional	“If you think that with everything you've had going on… that the trial is a bit too much for you… then we would say, we could draw a line…”
Active monitoring is your best option outside of the trial	Against	Health professional	“[The tumor is] all out and the risk of recurrence is less than half, so we would feel that your best bet is to have a very close observation…”
You need time to think before you decide	Against or unclear	Health professional	“You don't have to make a decision today… if you're even just wanting to consider it and go away and have a think…”

Informed by the analysis of the consultations, we analyzed the patient interviews and practitioner interviews thematically. In cases in which we had an audio‐recorded trial consultation and an associated patient and/or practitioner interview, we conducted integrative within‐case analysis 26. This involved drawing on themes arising from the consultations to produce narratives for each case describing how patients and practitioners viewed and responded to communication in consultations, and thereby linking the analysis of the consultations and interviews.

F.C.S. led all aspects of the analysis, with S.L.B. and B.Y. also reading a subset of transcripts and meeting periodically with F.C.S. to develop and refine the analysis. QSR Nvivo 11 27 was used to organize the data set and assist the analysis process. Illustrative quotes from the results are listed in Tables 3 and 4, with associated quote identifiers (e.g., Q1) listed in the results.

**Table 3:**
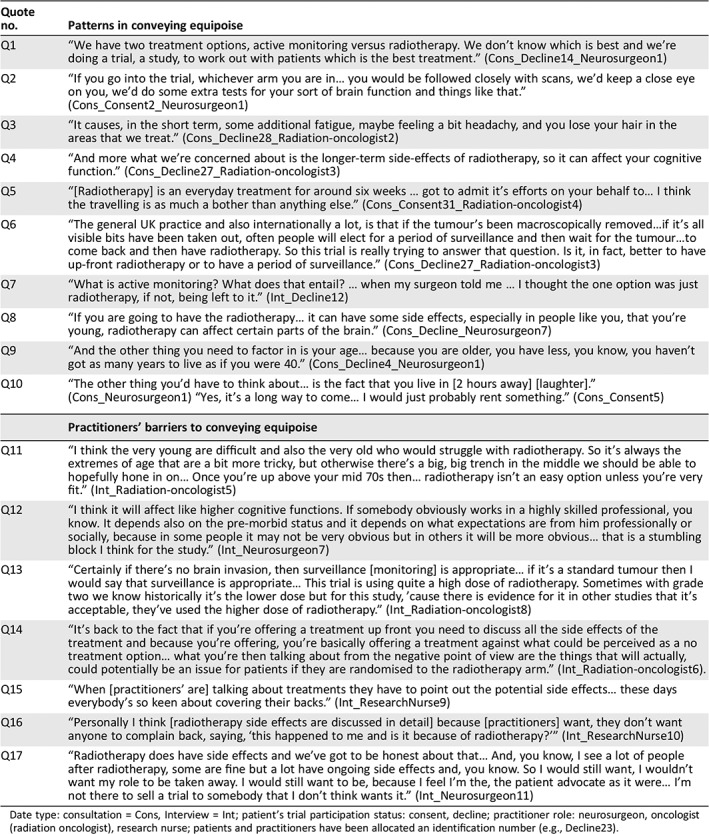
Quotes to illustrate patterns and barriers in conveying equipoise

Quote no.	Patterns in conveying equipoise
Q1	“We have two treatment options, active monitoring versus radiotherapy. We don't know which is best and we're doing a trial, a study, to work out with patients which is the best treatment.” (Cons_Decline14_Neurosurgeon1)
Q2	“If you go into the trial, whichever arm you are in… you would be followed closely with scans, we'd keep a close eye on you, we'd do some extra tests for your sort of brain function and things like that.” (Cons_Consent2_Neurosurgeon1)
Q3	“It causes, in the short term, some additional fatigue, maybe feeling a bit headachy, and you lose your hair in the areas that we treat.” (Cons_Decline28_Radiation‐oncologist2)
Q4	“And more what we're concerned about is the longer‐term side‐effects of radiotherapy, so it can affect your cognitive function.” (Cons_Decline27_Radiation‐oncologist3)
Q5	“[Radiotherapy] is an everyday treatment for around six weeks … got to admit it's efforts on your behalf to… I think the travelling is as much a bother than anything else.” (Cons_Consent31_Radiation‐oncologist4)
Q6	“The general UK practice and also internationally a lot, is that if the tumour's been macroscopically removed…if it's all visible bits have been taken out, often people will elect for a period of surveillance and then wait for the tumour…to come back and then have radiotherapy. So this trial is really trying to answer that question. Is it, in fact, better to have up‐front radiotherapy or to have a period of surveillance.” (Cons_Decline27_Radiation‐oncologist3)
Q7	“What is active monitoring? What does that entail? … when my surgeon told me … I thought the one option was just radiotherapy, if not, being left to it.” (Int_Decline12)
Q8	“If you are going to have the radiotherapy… it can have some side effects, especially in people like you, that you're young, radiotherapy can affect certain parts of the brain.” (Cons_Decline_Neurosurgeon7)
Q9	“And the other thing you need to factor in is your age… because you are older, you have less, you know, you haven't got as many years to live as if you were 40.” (Cons_Decline4_Neurosurgeon1)
Q10	“The other thing you'd have to think about… is the fact that you live in [2 hours away] [laughter].” (Cons_Neurosurgeon1) “Yes, it's a long way to come… I would just probably rent something.” (Cons_Consent5)
	**Practitioners’ barriers to conveying equipoise**
Q11	“I think the very young are difficult and also the very old who would struggle with radiotherapy. So it's always the extremes of age that are a bit more tricky, but otherwise there's a big, big trench in the middle we should be able to hopefully hone in on… Once you're up above your mid 70s then… radiotherapy isn't an easy option unless you're very fit.” (Int_Radiation‐oncologist5)
Q12	“I think it will affect like higher cognitive functions. If somebody obviously works in a highly skilled professional, you know. It depends also on the pre‐morbid status and it depends on what expectations are from him professionally or socially, because in some people it may not be very obvious but in others it will be more obvious… that is a stumbling block I think for the study.” (Int_Neurosurgeon7)
Q13	“Certainly if there's no brain invasion, then surveillance [monitoring] is appropriate… if it's a standard tumour then I would say that surveillance is appropriate… This trial is using quite a high dose of radiotherapy. Sometimes with grade two we know historically it's the lower dose but for this study, 'cause there is evidence for it in other studies that it's acceptable, they've used the higher dose of radiotherapy.” (Int_Radiation‐oncologist8)
Q14	“It's back to the fact that if you're offering a treatment up front you need to discuss all the side effects of the treatment and because you're offering, you're basically offering a treatment against what could be perceived as a no treatment option… what you're then talking about from the negative point of view are the things that will actually, could potentially be an issue for patients if they are randomised to the radiotherapy arm.” (Int_Radiation‐oncologist6).
Q15	“When [practitioners’ are] talking about treatments they have to point out the potential side effects… these days everybody's so keen about covering their backs.” (Int_ResearchNurse9)
Q16	“Personally I think [radiotherapy side effects are discussed in detail] because [practitioners] want, they don't want anyone to complain back, saying, ‘this happened to me and is it because of radiotherapy?’” (Int_ResearchNurse10)
Q17	“Radiotherapy does have side effects and we've got to be honest about that… And, you know, I see a lot of people after radiotherapy, some are fine but a lot have ongoing side effects and, you know. So I would still want, I wouldn't want my role to be taken away. I would still want to be, because I feel I'm the, the patient advocate as it were… I'm not there to sell a trial to somebody that I don't think wants it.” (Int_Neurosurgeon11)

Date type: consultation = Cons, Interview = Int; patient's trial participation status: consent, decline; practitioner role: neurosurgeon, oncologist (radiation oncologist), research nurse; patients and practitioners have been allocated an identification number (e.g., Decline23).

**Table 4:**
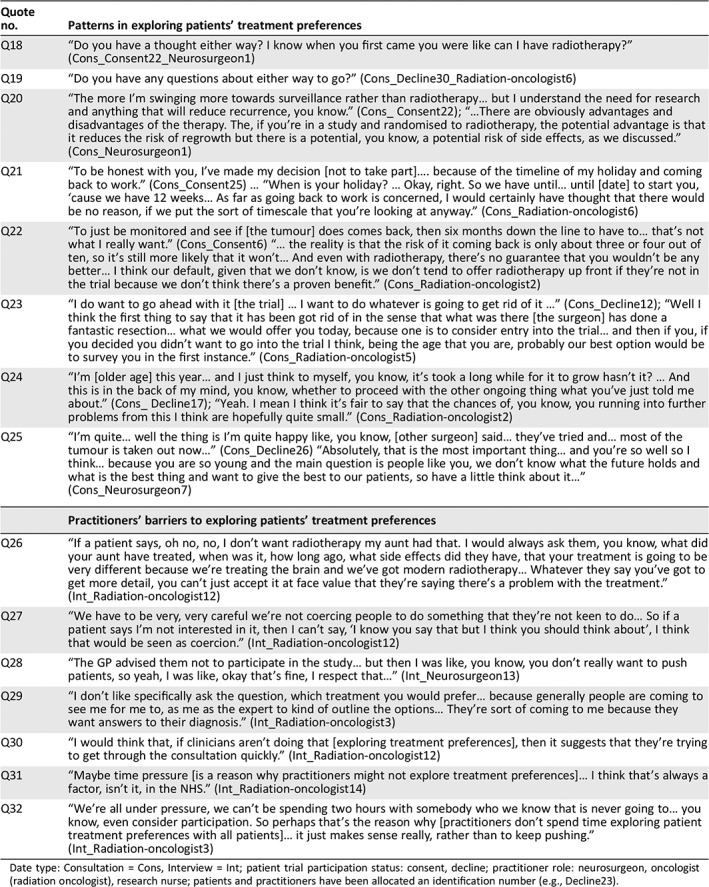
Quotes to illustrate patterns and barriers to exploring patients’ treatment preferences

Quote no.	Patterns in exploring patients’ treatment preferences
Q18	“Do you have a thought either way? I know when you first came you were like can I have radiotherapy?” (Cons_Consent22_Neurosurgeon1)
Q19	“Do you have any questions about either way to go?” (Cons_Decline30_Radiation‐oncologist6)
Q20	“The more I'm swinging more towards surveillance rather than radiotherapy… but I understand the need for research and anything that will reduce recurrence, you know.” (Cons_ Consent22); “…There are obviously advantages and disadvantages of the therapy. The, if you're in a study and randomised to radiotherapy, the potential advantage is that it reduces the risk of regrowth but there is a potential, you know, a potential risk of side effects, as we discussed.” (Cons_Neurosurgeon1)
Q21	“To be honest with you, I've made my decision [not to take part]…. because of the timeline of my holiday and coming back to work.” (Cons_Consent25) … “When is your holiday? … Okay, right. So we have until… until [date] to start you, ‘cause we have 12 weeks… As far as going back to work is concerned, I would certainly have thought that there would be no reason, if we put the sort of timescale that you're looking at anyway.” (Cons_Radiation‐oncologist6)
Q22	“To just be monitored and see if [the tumour] does comes back, then six months down the line to have to… that's not what I really want.” (Cons_Consent6) “… the reality is that the risk of it coming back is only about three or four out of ten, so it's still more likely that it won't… And even with radiotherapy, there's no guarantee that you wouldn't be any better… I think our default, given that we don't know, is we don't tend to offer radiotherapy up front if they're not in the trial because we don't think there's a proven benefit.” (Cons_Radiation‐oncologist2)
Q23	“I do want to go ahead with it [the trial] … I want to do whatever is going to get rid of it …” (Cons_Decline12); “Well I think the first thing to say that it has been got rid of in the sense that what was there [the surgeon] has done a fantastic resection… what we would offer you today, because one is to consider entry into the trial… and then if you, if you decided you didn't want to go into the trial I think, being the age that you are, probably our best option would be to survey you in the first instance.” (Cons_Radiation‐oncologist5)
Q24	“I'm [older age] this year… and I just think to myself, you know, it's took a long while for it to grow hasn't it? … And this is in the back of my mind, you know, whether to proceed with the other ongoing thing what you've just told me about.” (Cons_ Decline17); “Yeah. I mean I think it's fair to say that the chances of, you know, you running into further problems from this I think are hopefully quite small.” (Cons_Radiation‐oncologist2)
Q25	“I'm quite… well the thing is I'm quite happy like, you know, [other surgeon] said… they've tried and… most of the tumour is taken out now…” (Cons_Decline26) “Absolutely, that is the most important thing… and you're so well so I think… because you are so young and the main question is people like you, we don't know what the future holds and what is the best thing and want to give the best to our patients, so have a little think about it…” (Cons_Neurosurgeon7)
	**Practitioners’ barriers to exploring patients' treatment preferences**
Q26	“If a patient says, oh no, no, I don't want radiotherapy my aunt had that. I would always ask them, you know, what did your aunt have treated, when was it, how long ago, what side effects did they have, that your treatment is going to be very different because we're treating the brain and we've got modern radiotherapy… Whatever they say you've got to get more detail, you can't just accept it at face value that they're saying there's a problem with the treatment.” (Int_Radiation‐oncologist12)
Q27	“We have to be very, very careful we're not coercing people to do something that they're not keen to do… So if a patient says I'm not interested in it, then I can't say, ‘I know you say that but I think you should think about’, I think that would be seen as coercion.” (Int_Radiation‐oncologist12)
Q28	“The GP advised them not to participate in the study… but then I was like, you know, you don't really want to push patients, so yeah, I was like, okay that's fine, I respect that…” (Int_Neurosurgeon13)
Q29	“I don't like specifically ask the question, which treatment you would prefer… because generally people are coming to see me for me to, as me as the expert to kind of outline the options… They're sort of coming to me because they want answers to their diagnosis.” (Int_Radiation‐oncologist3)
Q30	“I would think that, if clinicians aren't doing that [exploring treatment preferences], then it suggests that they're trying to get through the consultation quickly.” (Int_Radiation‐oncologist12)
Q31	“Maybe time pressure [is a reason why practitioners might not explore treatment preferences]… I think that's always a factor, isn't it, in the NHS.” (Int_Radiation‐oncologist14)
Q32	“We're all under pressure, we can't be spending two hours with somebody who we know that is never going to… you know, even consider participation. So perhaps that's the reason why [practitioners don't spend time exploring patient treatment preferences with all patients]… it just makes sense really, rather than to keep pushing.” (Int_Radiation‐oncologist3)

Date type: Consultation = Cons, Interview = Int; patient trial participation status: consent, decline; practitioner role: neurosurgeon, oncologist (radiation oncologist), research nurse; patients and practitioners have been allocated an identification number (e.g., Decline23).

## Results

### Participants’ and Qualitative Data Set Characteristics

Table 5 summarizes key participant and data set characteristics. Data were collected on 40 patients and 18 practitioners from 13 U.K. sites. These comprised 43 audio‐recorded trial consultations (37 initial and 6 subsequent consultations) collected from 39 patients, and 18 practitioner and 23 patient interviews. For 22 of 40 patients, we captured all three linked data types: trial consultation, patient interview, and associated practitioner interview. Of the 18 practitioners interviewed, 11 had at least one recorded trial consultation. Most patients were female (*n* = 23, 58%), and median age was 57 years (range, 29–78). Of those recruited from England (*n* = 35), 26% (*n* = 9) were from the most deprived areas, 37% (*n* = 13) were from areas of average deprivation, and 37% were from the least deprived areas (*n* = 13). The overall consent rate to ROAM/EORTC‐1308 at the time of writing is 28%. Of the patients who participated in the qualitative study, approximately half also participated in ROAM/EORTC‐1308 (*n* = 19/40, 48%).

**Table 5:**
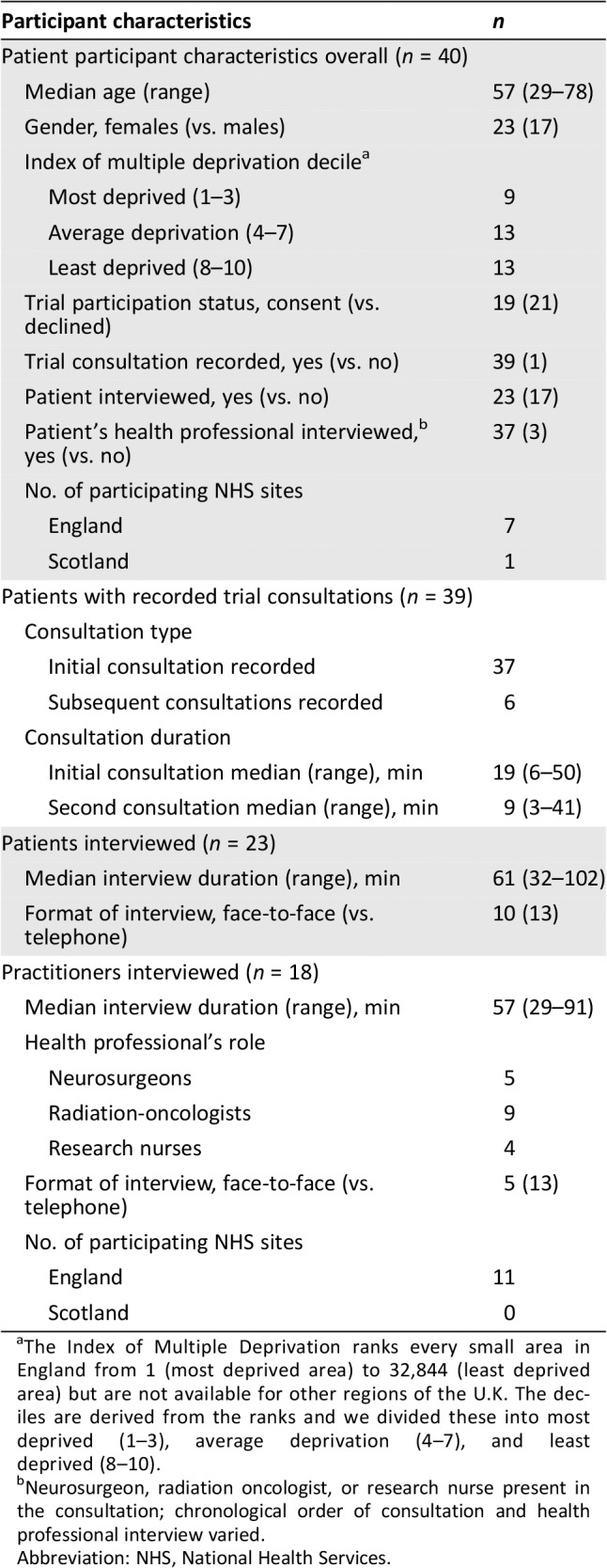
Summary of participant and data characteristics

Participant characteristics	*n*
Patient participant characteristics overall (*n* = 40)	
Median age (range)	57 (29–78)
Gender, females (vs. males)	23 (17)
Index of multiple deprivation decile^a^	
Most deprived (1–3)	9
Average deprivation (4–7)	13
Least deprived (8–10)	13
Trial participation status, consent (vs. declined)	19 (21)
Trial consultation recorded, yes (vs. no)	39 (1)
Patient interviewed, yes (vs. no)	23 (17)
Patient's health professional interviewed,^b^ yes (vs. no)	37 (3)
No. of participating NHS sites	
England	7
Scotland	1
Patients with recorded trial consultations (*n* = 39)	
Consultation type	
Initial consultation recorded	37
Subsequent consultations recorded	6
Consultation duration	
Initial consultation median (range), min	19 (6–50)
Second consultation median (range), min	9 (3–41)
Patients interviewed (*n* = 23)	
Median interview duration (range), min	61 (32–102)
Format of interview, face‐to‐face (vs. telephone)	10 (13)
Practitioners interviewed (*n* = 18)	
Median interview duration (range), min	57 (29–91)
Health professional's role	
Neurosurgeons	5
Radiation‐oncologists	9
Research nurses	4
Format of interview, face‐to‐face (vs. telephone)	5 (13)
No. of participating NHS sites	
England	11
Scotland	0

a The Index of Multiple Deprivation ranks every small area in England from 1 (most deprived area) to 32,844 (least deprived area) but are not available for other regions of the U.K. The deciles are derived from the ranks and we divided these into most deprived (1–3), average deprivation (4–7), and least deprived (8–10).

b Neurosurgeon, radiation oncologist, or research nurse present in the consultation; chronological order of consultation and health professional interview varied.

Abbreviation: NHS, National Health Services.

### Qualitative Results

#### *Patterns in Conveying Clinical Equipoise*

In consultations, all practitioners emphasized clinical equipoise by describing the lack of evidence and the uncertainty as to whether patients should be actively monitored or receive radiotherapy following surgery (Table 3, Q1). They often presented ROAM/EORTC‐1308 as an opportunity for patients to receive additional care, regardless of the treatment to which they were allocated (Q2). Some patients described this as a motivation for participating.

However, as consultations progressed, practitioners described the treatment arms in ways that were unbalanced, indicating that they lacked equipoise. They regularly emphasized the risks of radiotherapy, giving details of potential side effects such as fatigue, hair loss (Q3), and cognitive impairment (Q4), and its inconveniences, such as travel or delay in returning to normal routine (Q5). Although some elaborated on the potential effect of radiotherapy in preventing meningioma regrowth, most only mentioned it briefly and focused on the side effects. Furthermore, the nature and degree of side effects were often not discussed, leading some patients to assume side effects were severe. Simultaneously, many practitioners neglected details about the process and risks of active monitoring (Q6). In a context in which active monitoring was typically the standard treatment pathway outside the trial at most sites, the pattern of emphasizing the drawbacks of radiotherapy, but not its potential benefits, and neglecting the drawbacks of active monitoring, was, in effect, an argument against participating in ROAM/EORTC‐1308. We found that patients, when interviewed, were particularly confused about the monitoring arm, and some were unaware that ROAM/EORTC‐1308 had two treatment arms (Q7). Although the patient information sheet referred to “active monitoring” to label this arm, and this term was also recommended in feedback to practitioners, practitioners often used terms such as, “observation,” “surveillance,” and “watch and wait,” which can have negative connotations for patients.

Practitioners often personalized their statements about the risks of radiotherapy to individual patients. For example, in consultations with younger patients, some practitioners commented that the risk of cognitive impairment from radiotherapy was greater for younger patients (Q8). Similarly, in consultations with older patients, some practitioners commented that the benefits of radiotherapy might be negligible for older patients (Q9). When subsequently interviewed, such patients often indicated that these personalized arguments were highly salient and deterred them from participating. Practitioners also highlighted personalized inconveniences (e.g., travel) in consultations before patients had themselves identified it as important in their decision making (Q10).

#### *Reasons Why Practitioners Do Not Convey Clinical Equipoise*

When interviewed, practitioners acknowledged the importance of the ROAM/EORTC‐1308 research question, but they also described personal views about radiotherapy that corroborated the patterns of imbalanced trial communication we observed in consultations. They described concerns about treating patients toward the younger end of the age spectrum with radiotherapy because such patients had more years to live with any adverse cognitive sequelae. Conversely, they described concerns about treating patients toward the older end of the age spectrum with radiotherapy because older patients had relatively fewer years of life during which meningioma regrowth could occur and because they might struggle to tolerate radiotherapy (Q11). When interviewed, some practitioners voiced more idiosyncratic views against radiotherapy: one commented that radiotherapy was less suited to patients who had “highly skilled” occupations because the impact on their cognitive functioning would be more pronounced (Q12), whereas another was concerned that the radiation dose delivered in ROAM/EORTC‐1308 was higher than that which they would deliver outside the trial (Q13).

Practitioners acknowledged when interviewed that they provided patients with more information about radiotherapy than active monitoring but commented that it was essential to comprehensively describe the risks of radiotherapy, as patients would typically receive active monitoring outside of the trial (Q14). They also indicated that there could be ramifications for them personally if a patient subsequently complained about a side effect of radiotherapy that had not been detailed (Q15, Q16). Some practitioners commented that the trial diminished their clinical agency and they had a duty of care to provide patients with details of treatment risks, despite the lack of robust evidence on frequency and magnitude of these risks (Q17).

#### *Patterns in Exploring Patient Treatment Preferences*

In line with consultation feedback over the course of ROAM/EORTC‐1308, practitioners increasingly elicited patient treatment preferences (Table 4, Q18, Q19). Although it was rare, a few practitioners acknowledged patients’ reasons for their preferences and provided information to balance patients’ views of each arm to emphasize equipoise (Q20). Occasionally, practitioners responded to allay patient concerns about the process or inconvenience of radiotherapy. For example, when a patient indicated a preference for active monitoring during one consultation, the practitioner responded by exploring the patient's underlying reason for the preference. This identified that the patient had an unfounded concern that radiotherapy would delay their return to work. The practitioner's response regarding when and how radiotherapy could be delivered helped to allay the patient's concern, enabling them to participate without reservation (Q21).

More commonly, when patients voiced an interest in participating in ROAM/EORTC‐1308, practitioners prompted them to consider the possible downsides of radiotherapy (Q22, Q23), but they did not similarly prompt patients who expressed a preference for active monitoring (Q24, Q25) to consider the downsides of this management pathway. In this way, practitioners’ communication was loaded against radiotherapy and in favor of active monitoring. In effect, this worked against participation in ROAM/EORTC‐1308 because active monitoring was typically the standard treatment outside the trial in most sites.

#### *Reasons Why Practitioners Do Not Explore Patient Treatment Preferences*

Whereas some practitioners felt that exploring patient treatment preference was beneficial to discover patients’ preconceptions and address misconceptions (Q26), others feared that exploring treatment preferences could be viewed as tantamount to coercing patients to participate in ROAM/EORTC‐1308 (Q27). Some patients were also advised by practitioners outside of the trial (including neurosurgeons and family doctors) not to participate. Some worried that exploring patient treatment preferences when these had been informed by discussions with other practitioners could also be viewed as coercive (Q28). Two practitioners suggested that exploring treatment preferences conflicted with patient expectations of a practitioner's role, which they felt was to advise patients on which treatments were best for them (Q29). Practitioners alluded to time limitations during consultations as a barrier to exploring treatment preferences (Q30, Q31). Some suggested that it would be a poor use of time to explore treatment preferences with patients who appeared disinterested in the trial, as they were unlikely to participate (Q32).

## Discussion

This is the first study to examine how practitioners convey equipoise and respond to patient treatment preferences in a neuro‐oncology trial, like ROAM/EORTC‐1308 20, that compares markedly different management pathways. We anticipated that some practitioners might lack clinical equipoise and that this could influence their communication with patients and impede informed consent discussions and trial recruitment 6. In keeping with previous studies, practitioners expressed support for the trial question, but in consultations, they often lacked balance in how they presented the treatment arms and responded to patient preferences 9, 28. For example, we identified that practitioners’ responses were loaded against radiotherapy and favored active monitoring, often in ways that were personalized to a patient's individual characteristics. This effectively worked against trial participation and informed consent, particularly as personalized risk communication is more persuasive than generic risk communication 29.

**Figure 2:**
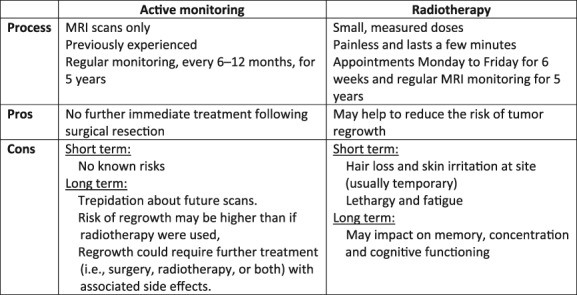
Recommendations on key pros and cons to convey to patients regarding the management pathways in ROAM/EORTC‐1308.Abbreviation: MRI, magnetic resonance imaging.

Our study indicates that practitioners working on neuro‐oncology trials that investigate a treatment (in this case radiotherapy) versus active monitoring face previously unreported challenges in conveying equipoise. Treatments like radiotherapy pose a risk of cognitive impairment, which threatens a patient's sense of identity 30 and which many fear more than death 31. This might partly explain why practitioners spent more time detailing the risks of radiotherapy, including cognitive impairment, than emphasizing the potential benefit of radiotherapy in reducing meningioma recurrence. Despite this focus on the radiotherapy risks in consultations, discussion of the likely degree of any impairment and how it might affect patients was often limited. In this lacuna, some patients assumed the impairment would be severe. Practitioners need to explain the potential drawbacks of both the intervention and active monitoring arms in such trials in ways that make sense to patients. Not doing so risks leaving patients with misunderstandings about trial treatments that can lead to them deciding to join or decline based on false premises 10, 32. Figure 2 provides example arguments for and against both management pathways that practitioners might convey and discuss with patients to further balance ROAM/EORTC‐1308 trial consultations. Conducting feasibility trials with embedded qualitative studies helps to identify and address such issues, enabling practitioners to optimize trial communication before a full‐scale trial 33. Additionally, in the context of trial recruitment consultations, in which patients have been assessed as eligible based on criteria specified in the trial protocol, we consider it advisable to avoid personalized risk communication across both treatment arms.

This is the first study to identify the reasons that underlie practitioner reluctance to explore patient treatment preferences during trial consultations more broadly. Although practitioners increasingly elicited treatment preferences throughout the course of ROAM/EORTC‐1308, suggesting that the feedback we provided influenced their communication, they remained reluctant to explore treatment preferences for fear that it could be viewed as coercive. This led them to largely accept patient treatment preferences at face value and avoid providing information to balance preferences. This is especially pertinent because the priorities that inform patients’ deliberations about treatments are rather different to those that inform the deliberations of oncologists and other practitioners 34. Practitioner reluctance to explore patient treatment preferences seemed to reflect the belief that doing so is inconsistent with patient voluntariness, founded on an assumption that voluntariness depends on independent choice 35. Yet, exploration of patient treatment preference can be ethically appropriate and enhance informed consent and trial recruitment 10, 11. Future practitioner training might usefully introduce such ethical conceptions of autonomy as relational 35 and consider how far preference exploration is consistent with such conceptions. Future research should also explore patient views on the acceptability of treatment preference exploration to establish whether they find it supportive or coercive.

A major strength of this study was the triangulation of findings from analysis of trial consultations and interviews with the practitioner and patient interviews. The method of analyzing trial consultations and practitioner interviews has been adopted elsewhere 18 but less often accompanied by analysis of patient interviews 36 and never previously has such work drawn on argumentation theory. This approach allowed us to identify patterns in trial communication, such as practitioners’ responses to patient treatment preferences, and in turn, identify how practitioners’ beliefs about treatments undermined recruitment 9.

Qualitative studies are characterized by smaller sample sizes, but in‐depth analysis generates valuable insights 37 that will be transferable to future oncology trials. Our patient and health professional interview samples encompassed multiple sites and comprised a diverse sample of participants from across the U.K. We provided ongoing feedback to practitioners based on the ongoing data analyses to enhance their communication. Although we did not formally evaluate the impact of this because of the rarity of atypical meningioma and infrequent recruitment consultations, as we note above, practitioners seemed to increasingly elicit treatment preferences over the course of the trial. As such, it is important to note that our study is not an investigation of naturalistic communication. We also cannot rule out that being audio recorded may have influenced communication during recruitment consultations; however, research in other contexts has found little evidence of such influence 38. Our study focused on U.K.‐based patients and practitioners, and research in other countries is needed to explore the wider transferability of our findings. We focused on communication between patients and practitioners, yet patients’ family members play an important supportive role and future work examining their role in recruitment consultations may be illuminating. Furthermore, because of the relative rarity of atypical meningioma, ROAM/EORTC‐1308 practitioners did not have regular opportunities to hone and refine their trial communication in response to qualitative feedback, and it was not possible to formally assess the effect of qualitative feedback on trial communication or recruitment rates. Nevertheless, practitioners reported that the consultation feedback helped them to improve communication in subsequent trial consultations.

## Conclusion

Our study adopted qualitative methods to triangulate consultations and patient and practitioner interviews to explore how practitioners communicated about an oncology trial with widely differing management pathways. Practitioners encountered challenges in balancing the content and presentation of treatment arms, often due to concerns that radiotherapy might entail further burden and side effects, such as cognitive impairment. They also described reluctance to explore patient treatment preferences, largely due to fear of coercing patients to participate, and time constraints in clinics. These concerns heavily influenced the communication of practitioners in ways that were effectively loaded against trial participation. The results can be used to inform training to optimize oncology trial communication, particularly neuro‐oncology trials and when trials compare markedly different treatment arms. Such training might benefit from exploring practitioners’ views of decision‐making voluntariness and coercion by introducing recent ethical conceptions of autonomy as relational.

## Author Contributions

**Conception/design:** Frances C. Sherratt, Stephen L. Brown, Brian J. Haylock, Priya Francis, Helen Hickey, Carrol Gamble, Michael D. Jenkinson, Bridget Young

**Provision of study material or patients:** Michael D. Jenkinson

**Collection and/or assembly of data:** Frances C. Sherratt, Bridget Young

**Data analysis and interpretation:** Frances C. Sherratt, Stephen L. Brown, Michael D. Jenkinson, Bridget Young

**Manuscript writing:** Frances C. Sherratt, Stephen L. Brown, Brian J. Haylock, Priya Francis, Helen Hickey, Carrol Gamble, Michael D. Jenkinson, Bridget Young

**Final approval of manuscript:** Frances C. Sherratt, Stephen L. Brown, Brian J. Haylock, Priya Francis, Helen Hickey, Carrol Gamble, Michael D. Jenkinson, Bridget Young

## Disclosures

The authors indicated no financial relationships.

## Supporting information

See http://www.TheOncologist.com for supplemental material available online.

Supplemental Appendices
