# Supplementary material for: Challenges Conveying Clinical Equipoise and Exploring Patient Treatment Preferences in an Oncology Trial Comparing Active Monitoring with Radiotherapy (ROAM/EORTC 1308)
Source: Oncologist. 2020 Feb 11;25(4):e691–700. doi: 10.1634/theoncologist.2019-0571 (PMC7160418; doi:10.1634/theoncologist.2019-0571)
Supplement: Supplementary file 1 — Supplemental Appendices [file ONCO-25-e691-s001.pdf]

The Oncologist
The official journal of the Society for Translational Oncology

Supplemental Appendix for:
Challenges conveying clinical equipoise and exploring patient treatment preferences in an oncology trial comparing active monitoring with radiotherapy (ROAM/EORTC 1308)
Bridget Young et al.

Appendix A: Example topic guide for practitioner interviews

- Reiterate aims and procedure of interview. *How trials like the ROAM trial can be a lot to understand for patients, so we're doing the ROAM Information Study to learn ways to improve how we communicate research to patients {provide overview of topics we will discuss}.*
- Reassure re confidentiality.
- Check recruiter is happy to continue with interview.

Background information:

1. Can you tell me what your professional role is within the NHS and what your role entails?
2. Can you also tell me what your role is in relation to the ROAM trial? Can you give me an overview of what your role in relation to the ROAM trial entails?
3. How long have you had this role for in relation to the ROAM trial? How many patients have you discussed the ROAM trial with/approached about the trial so far? How many other trials have you worked on or recruited to?
4. Roughly how much of your time every six months would you say is spent recruiting patients to the ROAM trial?
5. How much of your time every six months is usually spent recruiting patients to other studies, other than the ROAM trial?

Discussing the trial:

6. In terms of care/treatment what will have happened to the patient by the point they get to discuss the ROAM trial with you? Will they have had any conversations about other research studies by that point?
7. Focusing on the ROAM trial specifically, could you tell me about the discussions you've had with patients so far? *Focusing on one particular discussion.* How has that been from your perspective?
8. I'm trying to build up a picture of what these discussions are like - who would be present/involved? What roles would different staff play in that discussion? Is there someone to help you get everything prepared for a patient coming in who's eligible?
9. How much would you know about the patient before the ROAM trial discussion? Will you have met them before? Is that prior knowledge (or lack of) something that you consider helpful or important?
10. So my understanding is that you'll see patients who are eligible for the ROAM trial fairly infrequently. Does that make a difference? What sort of things do you do before you see an eligible patient? Do you think through beforehand what you will say to patients about the ROAM trial?
11. At the start of the discussion, what would you say to the patient? Based on your most recent discussion, how did the patient look/respond at that initial point?

12. Again, I'm trying to build up a picture – would you walk me through what happens after that opening? What other topics do you cover next? What other topics do you think are important to discussion? *Prompt short/long term patient involvement.*
13. Is there anything that you find particularly difficult to explain about the ROAM trial or that you found a patient may struggle with? Did the patient(s) you saw ask many questions? What about? What are the easier aspects to explain?
14. Is there anything that you feel would help to make discussing the ROAM trial with patients more straightforward or make discussing the trial with patients a bit easier?
15. All patients in the ROAM trial have surgery first and then the trial involves allocation to either radiotherapy or active monitoring. Can you walk me through how you would explain this aspect? What was it like discussing the two different trial arms? Did you find that the patient appeared to prefer one of these arms over the other? If so, why did you think that was? How did you respond/manage this in the trial discussion? Did you find that the patient had a good understanding of the trial arms? *If no, prompt for what didn't they understand, whether the patient's view appeared to change regarding trial arms following discussion, and what was explained.(Explore treatment preferences in greater detail following preliminary analysis i.e.* if they've used it and what they think of it, and whether they think patients find it helpful?)
16. How do you approach the questions or 'uncertainties' that the ROAM trial can raise (e.g. naturally trials are done when there's a lack of evidence about what is the best treatment). How did the patient respond to this uncertainty? Do you find this a difficult concept for patients in any way? Do you think this would impact the way in which a patient views their relationship with you? If so, in what ways?
17. How did you find it best to bring the discussion about the ROAM trial to a close with the patient?
18. What did you think about having the discussion recorded? Do you think you might have discussed the trial differently in any ways if you were not being recorded? If so, in what ways?
19. Do you know of any other discussions the patient had about the ROAM trial with any other clinicians? Do you think that certain clinician roles are more suited to discuss certain aspects of the ROAM trial? If so, how and why might this be? Are you more comfortable describing some aspects compared to some other aspects? If so, which and why?

Patients:

20. Would you say you try to standardise the ROAM trial information for all patients or would you tailor the information in some way? Can you tell me how you decide what to tell different patients and why?
21. What do you hope to be the main messages that patients take away from the discussions that you have?
22. Do you think that the characteristics of some patients might make it easier or harder to discuss the ROAM trial? If so, what kind of characteristics? And in your experience are there also characteristics that differ between patients who say yes and those who say no to trials more generally? Do you think this affects the manner in which you approach patients about research and if so, how?
23. Considering the discussion you had, what factors on the whole do you think tended to be the main issues that they patient considered and influenced their decision? And on the whole, in your opinion, what factors do you think will be the main issues patients consider and influence patients' decisions to take part in the ROAM trial, overall?

24. We’ve already talked a bit about the sorts of questions patients ask? Is there anything else they ask about? How do you deal with these questions? How do you respond? How would you usually respond if you didn’t feel confident? Did the patient ask what you would do? Did they appear to have a treatment preference? How did you respond? *Focus particularly on treatment preferences, allocation, what trial involves.*
25. Based on the discussion(s) you have had, do you find that the timing of the trial discussion impacts on how patients respond to joining the trial? Is there any room to adjust the timing so that it is better for the patient? When do you think would be most appropriate time to discuss the trial during patients’ engagement with services?
26. Do you feel that patients have enough time to decide? Do you ever think that with more time patients might have made a different decision?

Trial participation:

27. Was there any point throughout the discussion(s) that you felt that the patient hadn’t really understood what’s being asked of them? How can you tell? Are there any steps that you take when that happens? Can you tell me about these?
28. Did you ever feel concerned that despite your best efforts, the patient had accepted or declined participation in the ROAM trial without achieving or seeking sufficient understanding? Is there anything that could have been done differently?
29. Do you ever come across patients that you are reticent (or reserved) about approaching? Could you tell me about this? What aspects might contribute towards this? What do you do?
30. Has there ever been a time when you have identified an eligible patient but thought that it was inappropriate to approach them? Are there any examples that you can tell me about? If not, have there ever been cases whereby you have identified an eligible patient and thought that it was inappropriate to approach the patient, but the patient was approached nevertheless? Please tell me more about this? For what reasons did you feel it might have been inappropriate?
31. Have you approached any participants that have declined to take part in ROAM? Are there any particular aspects that tend to put patients off? Can you tell me how you respond if a patient begins to signal that they are disinterested?
32. When a patient does decline, are you happy that it is an informed decision? Would you try to check that they understand what they are saying no to?
33. Contrary to this, could you tell me any aspects that you have found so far to encourage patients to participate in the ROAM trial? Why is this or why do you think this is?
34. Have you had contact with any patients that have withdrawn from the ROAM trial? If so, did they provide reasons for withdrawing? How did/would you respond to someone who is interested in withdrawing? (e.g. completion of some assessments)

General experience of approaching patients:

35. I just wondered, what your thoughts are on approaching patients for research in general. Are there any aspects of approaching patients for research you enjoy/find rewarding? What would you say the main challenges are? Is it something that you feel comfortable with? If not, what is it that makes you feel less comfortable?
36. Considering other research projects you have worked on more broadly, have you approached any participants that have declined? Are there any particular aspects that tend to put patients off medical research? Can you tell me how you respond if a patient begins to signal that they are disinterested?

37. Do you have much experience of patients withdrawing from trials? Are there any particular reasons that patients often attribute to withdrawing? Can you remember any previous discussions you've had (in relation to any other research projects) with a participant who is considering withdrawing from a trial? If so, how did this conversation go?
38. Some people have suggested that it can be difficult to approach patients when you are involved in their care because it may affect the patient-practitioner relationship. Is that something you have thought about in the past?

Information sheet and recruitment barriers:

39. How helpful do you think the information leaflets for patients are? What do you see as their main purpose?
40. Are there any ways that you think they could be improved? How do you think that would help the patients? What do you feel are the key features of the information leaflets or which aspects would you say are most helpful to you in recruiting patients?
41. Some trials struggle to recruit patients – what do you think are the main reasons for this?
42. Can you think of any ways to improve recruitment? What might make research more engaging for everyone involved but still with the safeguards for patients?
43. Are patients who are eligible for the ROAM trial also eligible for any other studies? Do you have any thoughts about the amount of research being conducted? Is research fatigue ever a consideration for the patients? For practitioners?

The practitioner and training:

44. Can you tell me a little about how you came to be involved in trial work?
45. What are your main reasons for being involved in research generally? And what do you find motivating or demotivating in respect to taking on a particular trial?
46. In relation to the ROAM trial more specifically, what do you find motivating or demotivating? Do you feel these views reflect those of your colleagues as well? Have your colleagues noted any motivating or demotivating aspects to the ROAM trial that you have not mentioned?
47. Do you think that your approach to research differs in any way to other practitioners in your role? Or do you think that it is fairly uniform across staff? In what ways?
48. How did you develop your approach to talking with patients about trials? Did you get a chance to sit in and observe colleagues recruiting before having to do it yourself? If so, what did you take away from that?
49. Have you ever had any specific training on recruiting patients to trials? Can you tell me a bit about what this involved? What were the key aspects you took from it? If not, do you think training of that sort would be helpful – in what ways?
50. What was your experience of training regarding the ROAM trial? Do you feel this was adequate? Which aspects stood out for you? Which aspects were you unsure of? In what ways do you think the training on the ROAM trial be improved?
51. In your opinion what key components should be included in a trial training programme?
    Are there any areas that could benefit from more / less focus considering current guidance?
52. What would you say is the preferred format for presenting the findings of the ROAM Information Study? Media, F2F, PowerPoint?
53. I just wondered if you can tell me a little more about what you feel are the barriers / challenges to implementing study feedback?

54. What would you say are the drivers or facilitators to implementing study feedback?
55. In your opinion will study feedback optimise the recruitment process? How?
56. What do you think about your role as a doctor/nurse and that of a trial recruiter? Do you ever feel pulled in different directions? If so, in what ways? What do you think can be done to achieve a balance between good levels of informed consent and recruitment rates?
57. If you were advising a doctor/nurse who was quite new to trial recruitment, what would be the main tips or pointers you would give them?

Demographics

58. Age, gender, clinical role (recorded at start), and number of years trials experience.

Is there anything else that you think is important to mention?

Thank you very much for your time.
